# Prevalence of SARS‐CoV2 in Communities Surrounding Bwindi–Mgahinga Mountain Gorilla Conservation Area, Uganda

**DOI:** 10.1002/puh2.70403

**Published:** 2026-10-04

**Authors:** Joseph M. Kungu, Joan Neumbe, Dan Brian Kiganira, Patience Tino, Josephine Namayanja, Ronald Kassuja, Annet Nankya, John B. Nizeyi

**Affiliations:** ^1^ College of Veterinary Medicine Animal Resources and Biosecurity Makerere University Kampala Uganda; ^2^ Central Public Health Laboratories Ministry of Health Kampala Uganda; ^3^ Ministry of Agriculture Animal Industry and Fisheries Entebbe Uganda; ^4^ Uganda Virus Research Institute Entebbe Uganda

**Keywords:** conservation, coronavirus disease 2019 (COVID‐19), mountain gorillas, One Health, severe acute respiratory syndrome coronavirus 2 (SARS‐CoV2), Uganda

## Abstract

**Background:**

Mountain gorillas are endangered primates that share approximately 99% of their DNA with humans. Consequently, they may be susceptible to infection with severe acute respiratory syndrome coronavirus 2 (SARS‐CoV2), the virus that causes coronavirus disease 2019 (COVID‐19). Understanding the burden of infection of human populations in the surroundings of these endangered species is critical to assess the potential risk of spillover. This study aimed to determine the prevalence of SARS‐CoV2 and its associated risk factors in communities surrounding the Bwindi–Mgahinga Conservation Area in Uganda.

**Methods:**

A cross‐sectional study was conducted among 576 participants from the districts of Kanungu, Kisoro and Rubanda. Nasopharyngeal swab samples were collected and analysed using real‐time reverse transcription quantitative polymerase chain reaction (RT qPCR) at the Uganda Virus Research Institute. Demographic and behavioural data were collected using a structured questionnaire administered via KoboToolbox. Data analysis was performed using Stata (version 16) with cross‐tabulation to generate descriptive statistics of demographic characteristics, self‐reported non‐pharmaceutical interventions (NPIs) and SARS‐CoV2‐specific RNA status.

**Results:**

The overall prevalence of SARS‐CoV2 was 8.0%. Prevalence was highest in Kanungu (10.7%), followed by Kisoro (6.1%) and Rubanda (5.0%). Washing hands often was the only self‐reported NPI associated with SARS‐CoV2 prevalence (*X* ^2^ = 5.5, *p* = 0.02).

**Conclusion:**

SARS‐CoV2 was present among communities surrounding the Bwindi–Mgahinga Conservation Area. Strengthening adherence to preventive measures, such as hand hygiene, is essential to reduce transmission risk within communities and potential spillover to endangered mountain gorillas.

## Graphical abstract

COVID‐19 threatens endangered mountain gorilla populations due to close human contact. A cross‐sectional study (*n* = 576) in communities around Bwindi–Mgahinga Conservation Area found 8.0% prevalence of SARS‐CoV2. Frequent handwashing was a predictor for reduced prevalence of SARS‐CoV2. Strengthening prevention measures is critical to limit transmission and potential human–gorilla spillover.

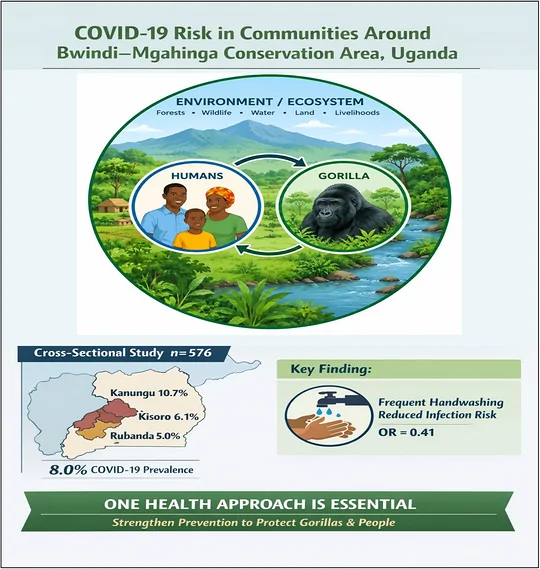

## 1 Background

The One Health approach recognizes the interconnectedness of human, animal, plant and environmental health [1, 2]. This framework is particularly relevant at the human–wildlife interface because over 70% of emerging and re‐emerging infectious diseases are zoonotic [3]. Globally, zoonoses account for an estimated 2.5 million illnesses and 2.7 million deaths annually, underscoring the risk of pathogen spillover [4]. Great apes, humans’ closest evolutionary relatives, are especially vulnerable to cross‐species transmission, including human respiratory pathogens [5, 6, 7].

Coronavirus disease 2019 (COVID‐19), caused by severe acute respiratory syndrome coronavirus 2 (SARS‐CoV2) of species *Betacoronavirus pandemicum* and family Coronaviridae, emerged in late 2019 and rapidly evolved into a global public health crisis. Declared a pandemic by the World Health Organization in March 2020, it resulted in over 60 million cases and 1.4 million deaths by the end of 2020, increasing to more than 260 million cases and 5 million deaths by 2021 [8, 9, 10]. Sub‐Saharan Africa reported a comparatively lower burden, partly due to limited testing, underreporting and younger population structures [11]. Uganda confirmed its first case on 21 March 2020 and implemented early non‐pharmaceutical interventions (NPIs), contributing to a slower initial spread [12]. By December 2020, the country had recorded 35,511 cases and 265 deaths [12, 13]. However, community transmission increased toward the end of 2020, with intensified waves in 2021 driven by more transmissible variants and limited early vaccine coverage [14].

The emergence of SARS‐CoV2 is linked to anthropogenic environmental changes, including habitat encroachment, deforestation and wildlife trade [4, 15, 16]. Initially considered a human health crisis, it has evolved into an anthropozoonotic threat, with potential transmission from humans to wildlife [17, 18]. Evidence of such transmission was documented in January 2021 when western lowland gorillas at the San Diego Zoo Safari Park contracted SARS‐CoV2 from an animal handler and developed respiratory illness before recovery [19].

In Uganda, the Bwindi–Mgahinga Conservation Area (BMCA) is a key habitat for the endangered mountain gorilla (*Gorilla beringei beringei*) [20]. Sharing approximately 99% genetic similarity with humans and possessing compatible viral receptors, these primates are highly susceptible to SARS‐CoV2 [18, 21]. Risk is heightened by habituation of gorilla groups for research and tourism [22]. The COVID‐19 pandemic further exacerbated this risk by disrupting local livelihoods, increasing poverty and driving illegal resource use within the park [21, 23]. These activities created unregulated human–forest interactions that bypass established infection prevention measures [24].

Despite the risk of spillover, data on SARS‐CoV2 prevalence in surrounding communities, the primary interface with the BMCA, remain limited. Assessing infection levels in these populations provides a critical sentinel measure of spillover risk, particularly as invasive diagnostics in gorillas are restricted to minimize harm [19]. This study, therefore, aimed to determine the prevalence of SARS‐CoV2 infection among communities surrounding the BMCA to inform public health strategies and safeguard both human populations and endangered mountain gorillas.

## 2 Materials and Methods

### 2.1 Study Area

The BMCA landscape encompasses communities surrounding Bwindi Impenetrable National Park and Mgahinga Gorilla National Park, bordering the Democratic Republic of the Congo to the west and Rwanda to the south (Figure 1) [25, 26, 27]. This region is characterized by densely populated rural communities whose livelihoods depend primarily on subsistence agriculture, small‐scale livestock keeping and tourism‐related activities. Most households cultivate crops, such as beans, potatoes, maize and bananas, on small landholdings. The terrain is mountainous and forms part of the Albertine Rift highlands, with elevations ranging from approximately 1200 to 2600 m above sea level. Limited infrastructure, high population density and reliance on natural resources shape the socio‐economic conditions of communities in this region [25, 27, 28].

**FIGURE 1:**
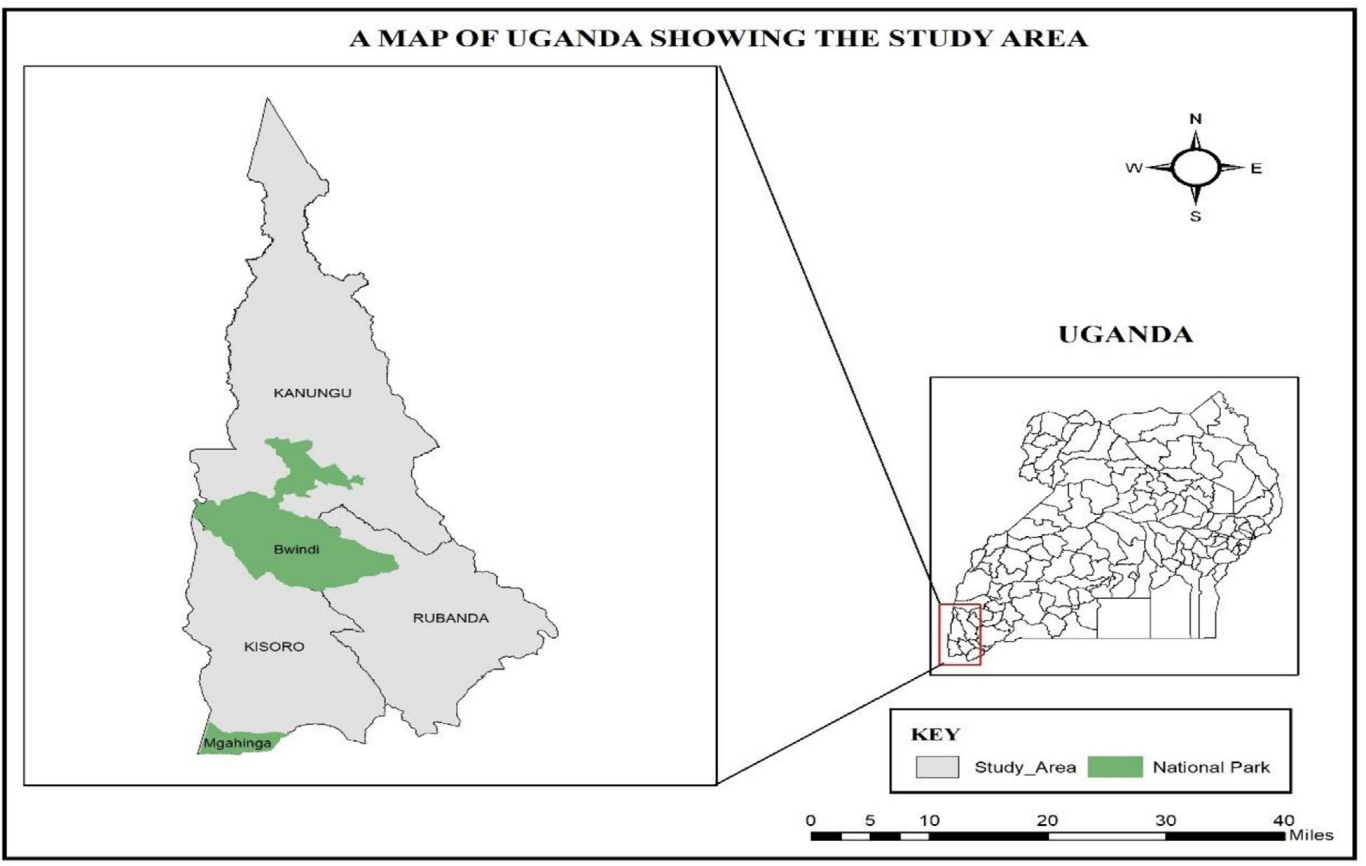
A map showing study sites.

Communities, living adjacent to protected areas, have frequent interactions with the forest ecosystem through livelihood activities, tourism employment and authorized or unauthorized access to forest resources. These interactions may increase contact between humans, wildlife and visitors, potentially influencing transmission dynamics of zoonotic and respiratory diseases. The study population included residents of villages located near the conservation areas across the three districts. The population is ethnically diverse, comprising indigenous Batwa people as well as Bakiga and Bafumbira ethnic groups. The Batwa historically depended on the forest for their livelihoods but now reside in settled communities around park boundaries following displacement from the forests [26, 27, 28].

### 2.2 Study Design

A cross‐sectional study was conducted between November and December 2021 to determine the prevalence of SARS‐CoV2 infection and associated predisposing factors among communities living in the BMCA landscape in southwestern Uganda. The study was carried out in Kisoro, Rubanda and Kanungu districts. Participants were selected using simple random sampling from household lists provided by community health workers in the study villages. Only one individual aged 10 years or older was selected from each household to represent the household in the survey. Upon visiting selected households, family members were asked to identify one willing individual to participate voluntarily.

### 2.3 Sample Size Determination

A sample size was determined using Kish Leslie formula [29]:

$$N=\frac{Z^{2}P\left(1-P\right)}{E^{2}}$$

where *N* is the required sample size, *Z* is the *z*‐score (1.96 for 95% confidence interval), *E* is the margin of error (0.05), and *P* is the expected population proportion. In the absence of prior prevalence data from this area, we assumed a conservative prevalence estimate of 50%, yielding a minimum required sample size of 385 participants. To increase precision and account for potential non‐response, we inflated the sample size to 576 participants at an expected response rate of 0.7 [30].

### 2.4 Data Collection

Data were collected using a piloted structured questionnaire programmed in KoboToolbox (https://kf.kobotoolbox.org) and administered using mobile phones. The questionnaire captured information on respondents’ demographic characteristics (age, sex, marital status, district of residence, religion, education level and occupation) and potential predisposing factors for SARS‐CoV2 infection, including adherence to five behavioural personal protective practices promoted by the Uganda Ministry of Health: frequent handwashing, consistent mask wearing, avoidance of hugging/kissing, coughing/sneezing into the elbow and avoiding touching the face.

Following questionnaire administration, a nasopharyngeal swab sample was collected from each participant by a trained research technician. Samples were placed in viral transport medium, stored at 2–8°C and transported to the Uganda Virus Research Institute (UVRI) for laboratory analysis.

### 2.5 Sample Processing and Analysis

Samples were analysed at the UVRI arbovirology laboratory through two major steps: RNA extraction and amplification. RNA extraction was performed using the QIAGEN QIAamp viral RNA kit following the manufacturer's instructions [31]. Amplification was performed using the Applied Biosystem real‐time quantitative PCR (ABI 7500 RT qPCR) system, as described by the manufacturer [32].

### 2.6 Data Analysis

Data from KoboToolbox were downloaded as an Excel file and imported into Stata version 16 for statistical analysis. Descriptive statistics (frequencies, percentages and means) were generated to characterize the study population. Cross‐tabulation using the chi‐square test was conducted to examine associations between demographic characteristics, self‐reported NPIs and SARS‐CoV2 infection status.

## 3 Results

### 3.1 Socio‐Demographic Characteristics of the Respondents

A total of 576 individuals participated in the study. The majority (72.7%) resided in Rubanda district. Females comprised slightly more than half of participants (53.5%), and the mean age was 34 years (range: 10–85 years). Farming was the primary occupation (66.7%), most participants had attained primary education (76.7%), and the majority were married (71.0%) and Christian (97.7%) (Table 1).

**TABLE 1:** Socio‐demographic characteristics of participants.

Variable	Category	Frequency	Percentage
Age	≤20 years	79	13.7
	20–29 years	133	23.1
	30–39 years	117	20.3
	40–49 years	92	16.0
	≥50 years	155	26.9
Gender	Female	308	53.5
	Male	268	46.5
Marital status	Married	409	71.0
	Not married	167	29.0
District	Kanungu	75	13.1
	Kisoro	82	14.2
	Rubanda	419	72.7
Religion	Christian	563	97.7
	Moslem	8	1.4
	Other	5	0.9
Education level	Primary	441	76.7
	Secondary	100	17.4
	Tertiary	34	5.9
Occupation	Business	90	15.6
	Farmer	384	66.7
	Civil servant	28	4.9
	Other	74	12.8

### 3.2 Prevalence of SARS‐CoV2 Around BMCA

Of the 576 nasal samples tested, 46 (8.0%; 95% CI: 5.9%–10.5%) were positive for SARS‐CoV2. Participants ≤20 years had the highest prevalence (11.4%), followed by those aged 20–29 years (5.3%), 30–39 years (5.1%), ≥50 years (5.2%) and 40–49 years (4.3%). Females had a higher prevalence (6.5%) than males (5.2%). Unmarried participants had higher prevalence (7.8%) compared to married participants (5.1%). Kanungu district recorded the highest prevalence (10.7%), followed by Kisoro (6.1%) and Rubanda (5.0%) (Table 2). Bivariate analysis revealed that among demographic characteristics, none showed statistically significant associations with SARS‐CoV2 prevalence. However, among personal protective practices, frequent handwashing was significantly associated with lower SARS‐CoV2 prevalence (*X* = 5.5, *p* = 0.02). Participants who reported washing hands often had a prevalence of 4.9% compared to 11.4% among those who did not (Table 2).

**TABLE 2:** Cross‐tabulation of demographic characteristics, personal protective practices and prevalence of severe acute respiratory syndrome coronavirus 2 (SARS‐CoV2) among participants.

Variable	Category	Total	SARS‐CoV2 positive (%)	*p* value
Age	≤20 years	79	9 (11.4)	0.28
	20–29 years	133	7 (5.3)	
	30–39 years	117	6 (5.1)	
	40–49 years	92	4 (4.3)	
	≥50 years	155	8 (5.2)	
Gender	Female	308	20 (6.5)	0.52
	Male	268	14 (5.2)	
Marital status	Married	409	21 (5.1)	0.22
	Not married	167	13 (7.8)	
District	Kanungu	75	8 (10.7)	0.16
	Kisoro	82	5 (6.1)	
	Rubanda	419	21 (5.0)	
Religion	Christian	563	34 (6.3)	0.66
	Moslem	8	0 (0)	
	Other	5	0 (0)	
Education level	Primary	441	23 (5.2)	0.14
	Secondary	100	10 (10.0)	
	Tertiary	34	1 (2.9)	
Occupation	Business	90	4 (4.4)	0.78
	Farmer	384	22 (5.7)	
	Civil servant	28	2 (7.1)	
	Other	74	6 (8.1)	
Wash hands often	Yes	487	24 (4.9)	**0.02**
	No	88	10 (11.4)	
Often put on mask	Yes	380	20 (5.3)	0.36
	No	196	14 (7.1)	
Often hug or kiss	Yes	305	21 (6.9)	0.30
	No	268	13 (4.9)	
Often cough or sneeze onto your elbow	Yes	151	12 (7.9)	0.21
	No	425	22 (5.2)	
Often touch your face	Yes	431	24 (5.6)	0.56
	Yes	145	10 (6.9)	

*Note:* Bold values significant *p* values <0.05.

## 4 Discussion

This study provides empirical evidence of SARS‐CoV2 circulation among human populations in the BMCA, highlighting an underexplored dimension of pandemic risk at the human–wildlife interface [18, 33, 34]. The observed prevalence of 8.0% aligns with national trends in Uganda during the study period, indicating that communities adjacent to protected areas were not epidemiologically isolated. In this setting, local communities, park staff and tourists form a continuous interface through which pathogens may be introduced into wildlife populations. Although direct evidence of SARS‐CoV2 infection in wild mountain gorillas remains limited due to constraints on invasive testing, detection in surrounding human populations underscores the need for precautionary surveillance [20, 28]. The present study, however, does not provide insights into circulating variants and transmission pathways of SARS‐Cov2, as genomic sequencing was not conducted.

The relatively lower prevalence compared to global estimates may reflect differences in epidemic timing, testing strategies and adherence to public health measures [10, 35, 36]. Uganda's early implementation of NPIs, including movement restrictions, mask use and public health campaigns, likely reduced transmission, particularly in rural areas [37, 38, 39, 40]. Reported adherence to measures, such as hand hygiene and mask use, combined with dispersed settlement patterns, may have further limited sustained transmission compared to urban settings [37, 41]. The self‐reported data on adherence to NPIs, however, were collected using subjective descriptors and may, however, be liable to abstract interpretation, recall and social desirability bias.

Spatial variation, with higher prevalence in Kanungu district, reflects cross‐border mobility. Its proximity to the Democratic Republic of Congo facilitates informal population movement, potentially undermining local containment efforts. These transboundary dynamics highlight the need for coordinated regional surveillance and response strategies [28, 42].

Age‐related differences, though not statistically significant, showed a higher prevalence among younger individuals, consistent with other studies. This group is typically more mobile and socially active, increasing exposure risk through trade, farming and tourism‐related activities, particularly where adherence to preventive measures is inconsistent [40, 43].

Moderate levels of human infection in the BMCA have disproportionate implications due to proximity to endangered mountain gorillas [3, 6, 24, 34]. The presence of SARS‐CoV2 in these communities reinforces concerns about anthropozoonotic spillover, given the close genetic similarity between humans and *G. beringei beringei*. Evidence of susceptibility of great apes to human respiratory pathogens, including documented SARS‐CoV2 infections in captive gorillas, supports this risk [7, 24, 34]. These emerging and re‐emerging infections result in negative economic implications as observed during the COVID‐19 outbreaks. The inactivity of the tourism industry led to loss of income by government of Uganda as well as the local people in BMCA communities [22, 28].

The public health and conservation significance of the 8.0% prevalence is further underscored by comparisons with other respiratory pathogens affecting great apes. Respiratory disease is the second leading cause of morbidity and mortality in habituated mountain gorillas [6, 7, 44]. Between 1990 and 2010, 18 outbreaks were reported in Rwanda, often affecting over 30% of group members and lasting weeks to months [45]. Documented spillover pathogens include human metapneumovirus, human respiratory syncytial virus and human rhinovirus C, with mortality reaching up to 20% in affected groups [33, 44, 46]. Although direct comparison across species requires caution, the presence of SARS‐CoV2 in edge communities, combined with this history of severe outbreaks, indicates a substantial spillover risk [6, 46, 47]. Unlike captive populations, wild gorillas lack access to treatment or vaccination, increasing potential impact [19].

From a One Health perspective, the BMCA represents a critical interface where human health, conservation and livelihoods intersect [3, 24]. Detection of SARS‐CoV2 in adjacent communities indicates a persistent risk of pathogen introduction into gorilla habitats, particularly through informal or unregulated forest interactions. Community‐level transmission may therefore represent a gap in existing conservation biosecurity measures [6, 24, 28, 34].

Strengthening integrated One Health surveillance systems that include both human and wildlife components is essential. Community‐based interventions, sustained infection prevention among park staff and visitors and targeted public health education could reduce cross‐species transmission risk. Supporting alternative livelihoods may further reduce high‐risk human–forest interactions exacerbated during the pandemic [3, 6].

## 5 Conclusion

This study indicates that SARS‐CoV2 was present among communities surrounding the BMCA. Although the observed prevalence was comparable to national trends, the proximity of these communities to endangered mountain gorillas presents a potential risk for cross‐species transmission. Strengthening adherence to preventive measures and implementing integrated One Health surveillance systems are essential to mitigate transmission risks at the human–wildlife interface.

## Author Contributions

**Joseph M. Kungu**: conceptualization, investigation, funding acquisition, writing – original draft, methodology, validation, visualization, writing – review and editing, software, formal analysis, supervision, resources, project administration, data curation. **Joan Neumbe**: investigation, writing – original draft, writing – review and editing, formal analysis, methodology, visualization, validation, data curation. **Dan Brian Kiganira**: writing – original draft, investigation, writing – review and editing, project administration, methodology, validation. **Patience Tino**: writing – original draft, investigation, writing – review and editing, project administration, resources. **Josephine Namayanja**: investigation, writing – original draft, methodology, validation, visualization, writing – review and editing. **Ronald Kassuja**: writing – original draft, investigation, visualization, validation, writing – review and editing. **Annet Nankya**: writing – original draft, investigation, writing – review and editing, methodology, formal analysis. **John B. Nizeyi**: conceptualization, funding acquisition, investigation, writing – original draft, methodology, validation, visualization, writing – review and editing, supervision, resources.

## Funding

The work was supported by the Government of Uganda through the Makerere University Research and Innovation Fund.

## Disclosure

The funders had no role in study design, data collection and analysis, decision to publish or preparation of the manuscript.

## Ethics Statement

This study was approved by Uganda Virus Research Institute Research Ethics Committee (UVRIREC‐2021‐12).

## Consent

Informed consent was obtained from all subjects and/or their legal guardian(s) before they participated in the study. Participants’ details were kept confidential by using special data codes. For participants unable to read, write or understand English, study information and consent statements were explained in the local language by a trained translator, and verbal informed consent was obtained before participation. Participants who could read and understand English provided written informed consent by signing the consent form. All procedures were conducted in compliance with COVID‐19 prevention guidelines. All methods were performed in accordance with the relevant guidelines and regulations of the journal.

## Conflicts of Interest

The authors declare no conflicts of interest.

## Data Availability

The datasets used and analysed during the current study are available on request.
